# Heterosis as Investigated in Terms of Polyploidy and Genetic Diversity Using Designed *Brassica juncea* Amphiploid and Its Progenitor Diploid Species

**DOI:** 10.1371/journal.pone.0029607

**Published:** 2012-02-21

**Authors:** Payal Bansal, Shashi Banga, S. S. Banga

**Affiliations:** Department of Plant Breeding and Genetics, Punjab Agricultural University, Ludhiana, India; Pennsylvania State University, United States of America

## Abstract

Fixed heterosis resulting from favorable interactions between the genes on their homoeologous genomes in an allopolyploid is considered analogous to classical heterosis accruing from interactions between homologous chromosomes in heterozygous plants of a diploid species. It has been hypothesized that fixed heterosis may be one of the causes of low classical heterosis in allopolyploids. We used Indian mustard (*Brassica juncea*, 2n = 36; AABB) as a model system to analyze this hypothesis due to ease of its resynthesis from its diploid progenitors, *B. rapa* (2n = 20; AA) and *B. nigra* (2n = 16; BB). Both forms of heterosis were investigated in terms of ploidy level, gene action and genetic diversity. To facilitate this, eleven *B. juncea* genotypes were resynthesized by hybridizing ten near inbred lines of *B. rapa* and nine of *B. nigra*. Three half diallel combinations involving resynthesized *B. juncea* (11×11) and the corresponding progenitor genotypes of *B. rapa* (10×10) and *B. nigra* (9×9) were evaluated. Genetic diversity was estimated based on DNA polymorphism generated by SSR primers. Heterosis and genetic diversity in parental diploid species appeared not to predict heterosis and genetic diversity at alloploid level. There was also no association between combining ability, genetic diversity and heterosis across ploidy. Though a large proportion (0.47) of combinations showed positive values, the average fixed heterosis was low for seed yield but high for biomass yield. The genetic diversity was a significant contributor to fixed heterosis for biomass yield, due possibly to adaptive advantage it may confer on de novo alloploids during evolution. Good general/specific combiners at diploid level did not necessarily produce good general/specific combiners at amphiploid level. It was also concluded that polyploidy impacts classical heterosis indirectly due to the negative association between fixed heterosis and classical heterosis.

## Introduction

Heterosis or hybrid vigour, manifested as improved performance of a hybrid in relation to its parents [1], arguably results from unique combinations of genetic and epigenetic information received from the parents. In spite of its enormous economic significance [2], genetic components of heterosis remain obscure [3]. Classical genetic explanations centre on genome wide dominance complementation or locus specific over dominance [4]. Both of these inter alia refer to non additive situations varying in degree. Epistatic interactions among non-allelic genes at different loci [5] are also considered to be a cause of complex phenotypic deviations that lead to heterosis. Most of the quantitative trait loci (QTL's) associated with heterosis and inbreeding depression in corn [6] and rice [7] show epistasis. However, in two different maize populations, QTL's for grain yield were almost always associated with a heterozygous genotype, indicating strong over dominance or pseudo-over dominance, with little evidence for epistasis [6], [8]. None of the current genetic models completely explain heterosis in polyploid plants as allelic and genomic dosage may have a greater role than the allelic complementation or interactions. Changes in dosage-dependent gene expression may be more profound than alteration in allelic interactions. In maize, the increased number of genes and the genome dosage seems to have a negative effect on growth and vigor, thereby increased levels of inbreeding depression [9]. Heterosis has also been explained in terms of quadratic function of the parental genetic distance (GD) at the underlying QTL's [10], [11]. All these genetic phenomenon of dominance, over dominance and epistasis are also considered to be generic features of gene regulatory networks [12]. Other theories to explain heterosis include ectopic or temporary alterations in gene expression levels in hybrids that deviate relative to mid parent proportion [12], [13], [14]. Most of the gene expression studies have, however, failed to establish any link between expression changes caused by heterozygosity and hybrid vigor [15], since such changes may also result from downstream molecular responses driven by heterotic growth effects and controlling genes [16], [17]. There is also ample genetic evidence which shows that alterations in the gene expression levels can affect phenotypes in quantitative manner [18], [19].

Aside its economic importance, heterosis has been an important biological contributor to crop evolution by conferring heterozygote advantage on naturally occurring hybrids [allopolyploids], leading to improved performance of adaptive traits like fecundity, viability and reaction to biotic and abiotic stresses [20]. This form of heterosis, also termed as fixed heterosis results from favorable interactions between the genes on their homoeologous genomes. It is analogous to classical heterosis, which primarily is a manifestation of interactions between homologous chromosomes in heterozygous plants of a diploid species. Contributions of fixed heterosis (level of ploidy) to classical heterosis or hybrid vigor in *Brassica* alloploids are unknown [21]. It is intriguing that comparatively a lower level of classical heterosis is expressed in several polyploids as compared to that reported in diploid progenitor species. It may be due to the negative effect of autoploidy on biomass production as observed in homozygous lines of *B. rapa* and *B. oleracea* [22]. A comparison of heterosis at two ploidy levels such as diploid and tetraploid in maize indicated that heterotic response of tetraploid maize lines differed significantly from that of diploid parents [1]. These contradict the concept of heterosis based on additive expression of differing alleles and its progression with ploidy.

Present studies were conceived to increase our understanding of the expression of heterosis in terms of genetic diversity, combining ability and polyploidy. Indian mustard (*Brassica juncea*, 2n = 36; AABB) was selected as the experimental material because of its economic importance as an oilseed crop and, the availability of hybrid breeding systems. It was also considered a suitable model system to analyze “fixed” heterosis because of the ease of its artificial resynthesis from desired genotypes of diploid parental species, *Brassica rapa* (2n = 20; AA) and *Brassica nigra* (2n = 16; BB). Understanding the basis of heterosis continues to be central to the attempts at imparting efficiency to the present method of identifying superior hybrid combinations.

## Results

**Figure 1:**
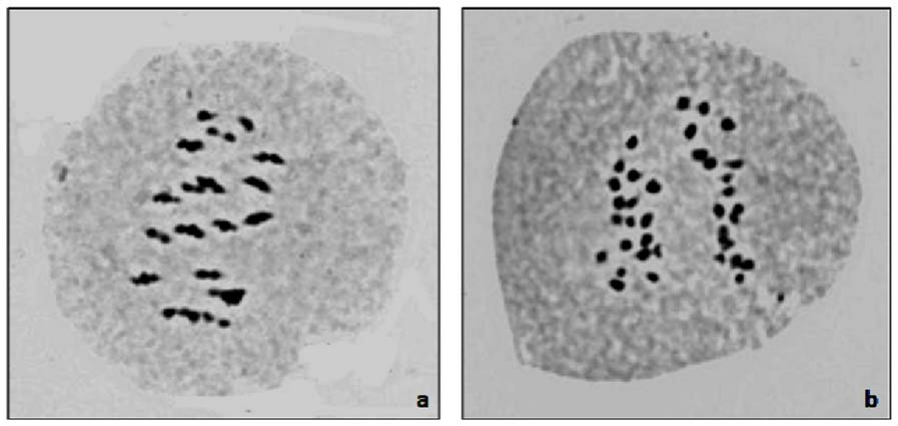
Meiotic configurations in resynthesized *Brassica juncea*. (a) 18 II during metaphase 1. (b) 18-18 separation during anaphase 1.

Eleven resynthesized *B.juncea* genotypes were developed using selected *B.rapa* and *B.nigra* near inbred lines (cf. Table 1) as parents. Synthetic *B.juncea* genotypes were named as SJN when *B.nigra* was used as female parent, and SJR when *B.rapa* was the female parent in the combination. The amphiploids had expected euploid chromosome number (2n = 36) with 18II as predominant meiotic configuration (cf. Figure 1a). Anaphase I mostly showed expected 18-18 separation (Figure 1b), indicating genomic stability of rsynthesized *Brassica juncea*. Erratic meiotic configurations were also observed, *albeit*, at lower frequencies. Pollen fertility in resynthesized *B. juncea* types was normal (>80%). A_1_ plants had lower seed set, which improved significantly following selfing.

**Table 1:** Pedigree record of resynthesized *B. juncea.*

Genotype code	Combination	
*B. juncea*	*B. rapa*	*B. nigra*
SJR 2	Sunford	PBn14
SJR 6	Sunbeam	PBn13
SJR 17	TCN-01	PBn16
SJR 23	Sanya	PBn16
SJR 57	Mitra	PBn13
SJR 59	EC 513427	PBn22
SJR 63	CH 1	PBn7
SJR 76	EC 513427	PBn19
SJR 113	EC 3390-101	PBn20
SJN 8	EC 513426	PBn17
SJN 14	Torch	PBn12

### Genetic diversity in resynthesized *B. juncea* and donor parents

**Figure 2:**
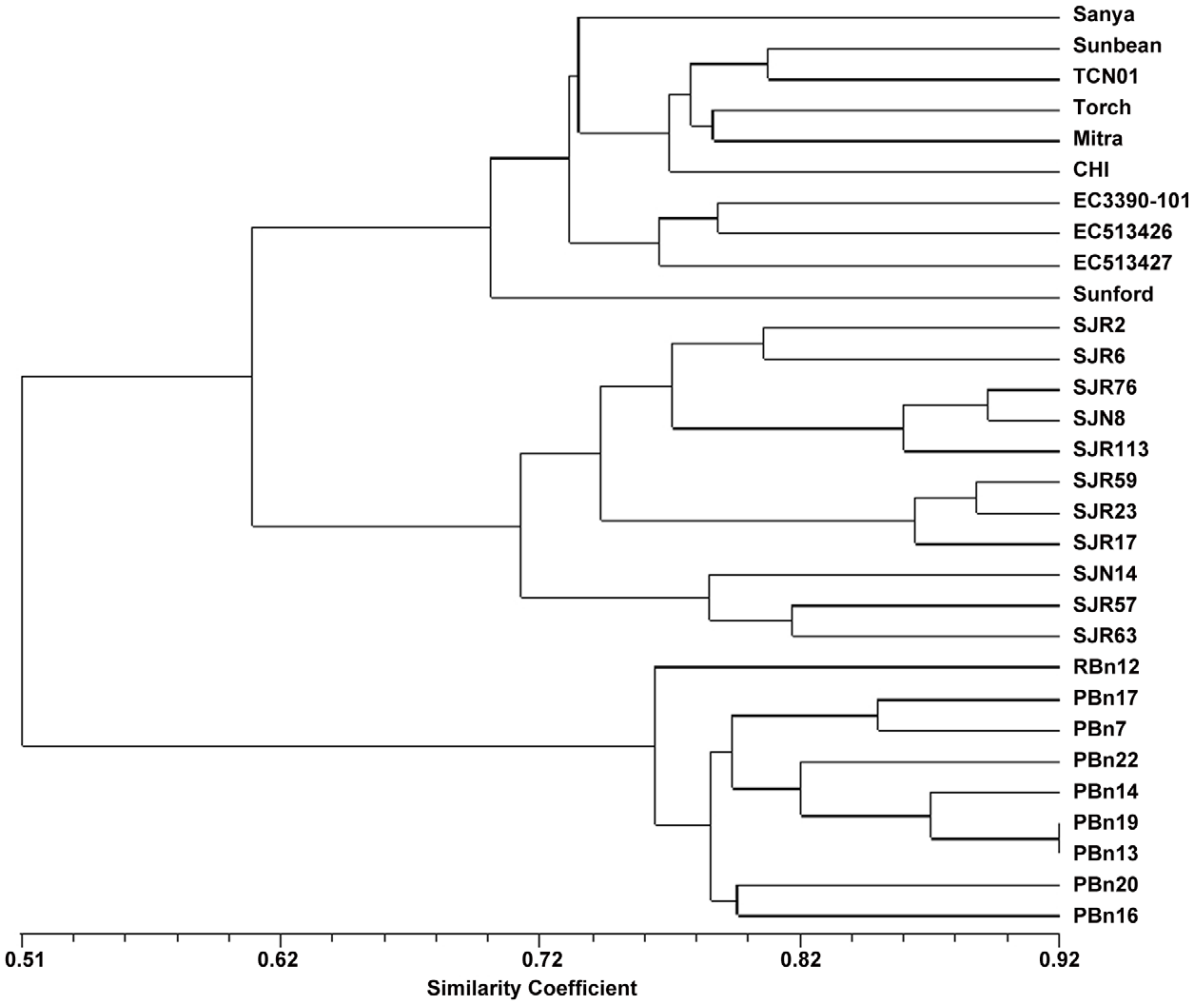
Dendrogramme showing molecular diversity in resynthesized *Brassica juncea* parents and natural *B. juncea*.

Resynthesized *B. juncea* genotypes (A_3_ generation) were assayed for genetic diversity using polymorphism generated by selected SSR primers in resynthesized *B. juncea* in comparison to the diploid progenitor species. The number of amplified bands ranged from 2–12 with polymorphic alleles also varying from 2–12. The PIC values ranged from 0.20 to 0.89; majority of the primers had PIC value in excess of 0.60 suggesting high informative content of the primers utilized. The genotypes belonging to two progenitor species occupied two extremes of dendrogram (cf. Figure 2). A-genome donor *B. rapa* had an overall dissimilarly coefficient of 0.40 with the resynthesized *B. juncea* group. *B. nigra* accessions were more diverse collectively and these related to *B. rapa* and resynthesized *B. juncea* with a mean similarly coefficient of 0.51. At a similarity coefficient of 0.4, three broad groups were resolved in resynthesized *B. juncea*. The group 1 comprised SJN 14, SJR 57 and SJR 63. Of these three, SJN 14 was most diverse. The group 1 differed from other two groups at the dissimilarity coefficient of 0.29. The group 2 was also assigned three genotypes namely SJR 17, SJR 23 and SJR 59. Of these SJR 23 and SJR 59 were very close and had a similarity coefficient of 0.88. The third major group comprising five genotypes had two subgroups with dissimilarity coefficient of 0.23. The subgroup-1 had three genotypes SJR 76, SJN 8 and SJR 113. Of these SJR 76 and SJN 8 were very close with a similarity coefficient of 0.89. SJR 113 was related to other two genotypes at a similarity coefficient of 0.86. The subgroup-II of the major group 3 included two relatively diverse genotypes SJR 2 and SJR 6, with a similarity coefficient of 0.80.

### Heterosis in resynthesized *B. juncea* and corresponding diploid genome donor genotypes

**Figure 3:**
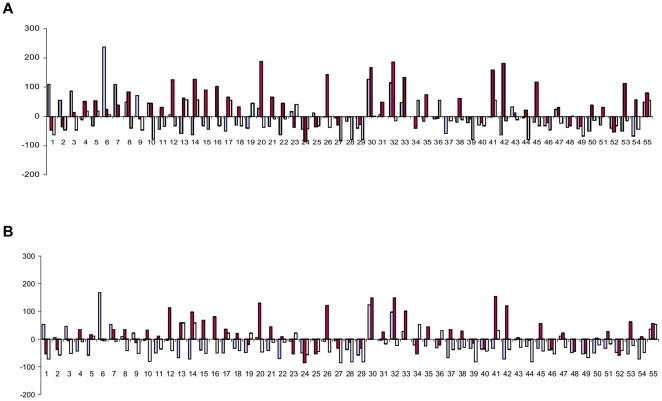
Pictorial representation of the manifestation of relative mid parent (A) and high parent (B) heterosis for seed yield in F_1_ hybrids of resynthesized *B. juncea* (red) and progenitor species, *B. rapa* (blue) and *B. nigra* (yellow).

**Figure 4:**
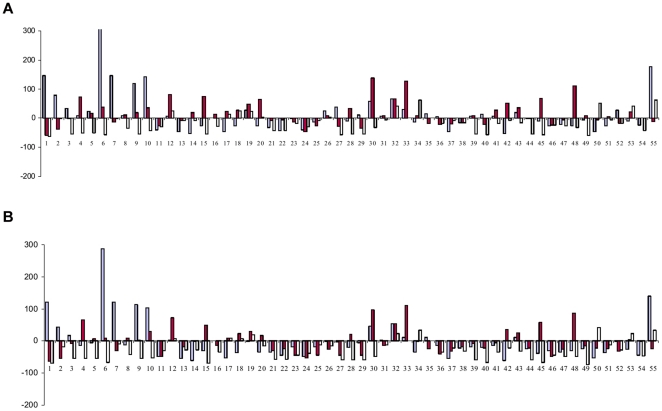
Pictorial representation of the manifestation of relative mid parent (A) and high parent (B) heterosis for biomass yield in F_1_ hybrids of resynthesized *B. juncea* (red) and progenitor species, *B. rapa* (blue) and *B. nigra* (yellow).

Fifty five F_1_ combinations, developed by hybridizing 11 resynthesized *B. juncea* in a half diallel fashion, and their corresponding diploid genotypes of the monogenomic parents, *B. rapa* and *B. nigra* combinations were field evaluated for calculation of fixed and classical heterosis over mid parent (RMPH) and high parent (RHPH) values (cf. Table S1) for seed and biomass yield. For example, a resynthesized *B. juncea* combination SJR 2 (Sanford×PBn14)×SJR 46 (EC 513427×PBn22), was evaluated along with the corresponding hybrid combinations of *B. rapa* (Sanford×EC 513427) and *B. nigra* (PBn14×PBn22) . The relative mid parent heterosis (RMPH) for seed yield in resynthesized *B. juncea* ranged from −85.4 (SJN 8×SJR 76) to 188.9 (SJN 8×SJR 6) with an average of 44.0 per cent (cf. Figure 3A). Twenty F_1_ combinations showed significant and positive heterosis over the mid parent. There was a lack of correspondence between the heterosis values at allotetraploid level in comparison to those recorded at diploid level in parallel combinations. The values for relative high parent heterosis( RHPH) for seed yield are presented in Figure 3B. Lack of relatedness between heterosis at diploid level and at alloploid level was indicated for RHPH. RHPH in the resynthesized *B. juncea* combination SJN 8×SJR 6 was not reflected in the corresponding *B. rapa* and *B. nigra* combinations. The trend of RMPH and RHPH for biomass yield is presented in Figure 4A–B. The RMPH for biomass yield varied from −58.9 (SJR 2×SJR 59) to 137.7 per cent (SJR 6×SJN 14). Akin to seed yield, there was no relatedness between the heterosis observed for biomass yield in *B. juncea* and corresponding *B. rapa* and *B. nigra* hybrids. For RHPH, the resynthesized *B. juncea*, SJR 6 (Sunbean×PBn13)×SJR 57 (Mitra×PBn13) was the best combination showing 111.3 per cent heterosis . Incidentally both the resynthesized *B. juncea* parents had a common *B. nigra* genotype, PBn13 in their pedigree. The *B. rapa* combination Sunbean×Mitra had RHPH value of 12.0 per cent for biomass yield. Overall, the average values of heterosis for biomass yield were marginally higher in *B. rapa* as compared to those recorded for *B. nigra* and the digenomic *B. juncea*. There was no correlation between heterosis and genetic diversity in resynthesized *B. juncea* (cf. Table 2). The same was true for *B. rapa*. In *B. nigra*, however, negative correlation was recorded between genetic diversity and heterosis for biomass yield. For seed yield, no correlation appeared to exist between heterosis and genetic diversity in *B. nigra*.

**Table 2:** Association of genetic diversity with heterosis for seed and biomass yield in resynthesized *B. juncea* and diploid progenitor species (*B. rapa* and *B. nigra*).

Correlation GD	Heterosis	Species		
		*B. juncea*	*B. rapa*	*B. nigra*
Seed yield	AMPH	0.06	0.08	0.08
	RMPH	0.04	0.16	0.16
	AHPH	0.04	−0.05	−0.05
	RHPH	0.06	0.04	0.04
Biomass yield	AMPH	0.17	0.21	−0.19
	RMPH	0.16	0.21	−0.13
	AHPH	0.07	0.18	−0.36*
	RHPH	0.11	0.19	−0.27*
	n	55	46	38

### Gene action in resynthesized *B. juncea* and progenitor species

The inferences for gene action were obtained from diallel crosses involving resynthesized *B. juncea* genotypes and progenitor genotypes of *B. rapa* and *B. nigra*. Significant variation was indicated for both the traits evaluated in all the three species (cf. Table 3). Genotypic variation for seed yield in *B. nigra*, however, was non significant. Variations due to parents, hybrids and parents *vs.* hybrids were significant.

**Table 3:** Mean square values in analysis of variance for seed yield (SY) and biomass yield (BY) in diallel crosses of *B. juncea* and its progenitor species *B. rapa* and *B. nigra*.

Source of Variation	*B. juncea*			*B. rapa*			*B. nigra*		
	df	SY	BY	df	SY	BY	df	SY	BY
Replication	1	87.12	1192.44	1	2215.81**	25215.06**	1	46.57	8357.30
Treatments	65	603.93**	17733.67**	54	344.86**	16276.98**	44	181.61*	11389.09**
Parents	10	152.31*	20742.77**	9	402.42**	6016.12*	8	106.95	17951.08**
Hybrids	54	656.17**	17248.19**	44	340.37**	18488.12**	35	192.61*	9159.42**
Parents vs. Hybrids	1	2298.91**	13858.35*	1	24.46	11334.92*	1	393.92*	36931.55**
Error	65	70.76	3075.20	54	143.06	2623.59	44	96.38	3656.61

*Significant at P = 0.05;

**Significant at P = 0.01.

#### Combining ability (CA)

The analysis (cf. Table 4) revealed highly significant variances for general combining ability (GCA) and specific combining ability (SCA) for seed as well as biomass yield . The data regarding genetic components of variance in diallel cross are presented in Table 5. Preponderance of dominance was indicated by consistently higher σ^2^_s_ and σ^2^_D_ values. This was also reflected in low heritability in narrow sense(cf. Table 5). The results for general combining ability of the resynthesized *B. juncea* and progenitor genotypes revealed that only two resynthesized *B. juncea* genotypes, SJR 6 (Sunbean×PBn13) and SJR 57 (Mitra×PBn13) showed positive and significant values for GCA effects, remaining were poor combiners. For SJR 57 and SJR 6, the corresponding *B. rapa* (Sunbean/Mitra) and *B. nigra* parents (PBn13for both) were poor combiners for seed yield (cf. Table 6). For *B. rapa* and *B. nigra*, only Sanya and PBn19 respectively were good combiners. However, none of these appeared with high gca effects in any of the resynthesized *B.juncea*. For biomass yield, SJN 8, SJR 6, SJR 23 and SJR 76 were excellent general combiners (cf. Table 6). For *B. rapa*, Sanford and CHI were good combiners but none of these was involved in good combiners identified for biomass yield in resynthesized *B. juncea*. For *B. nigra*, PBn13, PBn12 were excellent general combiners with positive values. Of these two, only PBn13 was involved in resynthesis of a good general combiner, SRJ 6. Resynthesized *B. juncea* strain SJN 14, with a good general combiner *B. nigra* genotype, PBn12 as one of the parent, was a poor general combiner. Of the fifty five combinations evaluated for seed and biomass yield, twenty two combinations had significant positive values. Interestingly, except one combination, SJR 6×SJR 113 (Sunbean×PBn13)×(EC 101×PBn20), none of the corresponding *B. rapa* or *B. nigra* parent (s) showed positive sca effects. Only in SJR 6×SJR 113, the parental *B. rapa* strains, Sunbean and EC 101 possessed positive sca effects. For biomass yield, seven combinations showed positive sca effects. Analysis of corresponding crosses at diploid level, suggest results similar to those recorded for seed yield. That the sca effects at diploid level were not reflected at amphiploid level was apparent from the fact that none of *B. rapa* (Sanford×EC 513427, Sanford×Torch, Sanford×EC 513427, Sunbean×EC 101, Mitra×CHI) or *B. nigra* (PBn13×PBn7) combinations showing high sca were represented in any of hybrid combination of resynthesized *B. juncea* showing significant and positive specific combining ability effects for seed yield.

**Table 4:** Mean square values in analysis of variance for combining ability in resynthesized *B. juncea* and its progenitor species.

Source of Variation	*B. juncea*			*B. rapa*			*B. nigra*		
	df	SY	BY	df	SY	BY	df	SY	BY
GCA	10	179.57**	14191.12**	9	178.81*	10386.65**	8	97.89**	12284.93**
SCA	55	324.21**	7898.78**	45	171.16**	7688.86**	36	89.23**	4230.01**
Error	65	35.38	1537.60	54	71.53	1311.79	44	48.18	1828.33

*Significant at P = 0.05;

**Significant at P = 0.01.

**Table 5:** Genetic components in diallel crosses of resynthesized *B. juncea* and progenitor species *B. rapa* and *B. nigra*.

Component	Seed Yield			Biomass Yield		
	*B. juncea*	*B. rapa*	*B. nigra*	*B. juncea*	*B. rapa*	*B. nigra*
σ^2^_g_	11.09	8.94	4.52	973.35	756.24	950.59
σ^2^_s_	288.84	99.63	41.04	6361.18	6377.07	2401.68
σ^2^_e_	35.38	71.53	48.19	1537.60	1311.79	1828.33
σ^2^_a_	22.18	17.88	9.03	1946.69	1512.48	1901.20
σ^2^_D_	288.84	99.63	41.04	6361.18	6377.07	2401.68
σ^2^_P_	346.40	189.04	98.26	9895.48	9201.34	6131.21
h^2^ (ns)	0.06	0.09	0.09	0.19	0.16	0.31
h^2^ (bs)	0.90	0.62	0.51	0.84	0.86	0.70

**Table 6:** General Combining Ability effects in *B. juncea* and its two progenitor species for seed yield and biomass yield.

*B. juncea*			*B. rapa*			*B. nigra*		
Genotype(s)	Seed Yield	Biomass Yield	Genotype(s)	Seed Yield	Biomass Yield	Genotype(s)	Seed Yield	Biomass Yield
Sunford×PBn14	0.15	−21.86*	Sunford	0.61	69.75**	PBn14	0.65	−26.10*
EC513427×PBn22	1.34	−29.73**	EC513427^a^	−8.87**	−40.34**	PBn22	−1.80	−23.05
PBn17×EC523426	2.17	60.48**	EC523426	0.24	−1.99	PBn17	−5.10*	−30.11*
Sunbean×PBn13	3.62*	37.45**	Sunbean	2.19	5.38	PBn13^b^	0.16	29.54*
TCN01-18×PBn16	−2.18	−23.91*	TCN01-18	0.67	−0.77	PBn16^c^	−9.67	7.83
Sonja×PBn16	0.90	33.65**	Sonja	6.86**	12.09	PBn16^c^	−9.67	7.83
PBn12×Torch	−8.48**	−42.26**	Torch	−0.77	5.87	PBn12	1.33	65.16**
EC513427×PBn19	0.09	23.03*	EC513427^a^	−8.87**	−40.34**	PBn19	4.91*	10.09
EC3390-101×PBn20	−3.91*	−12.41	EC3390-101	−1.37	−7.52	PBn20	3.09	5.43
Mitra×PBn13	4.87**	−15.55	Mitra	0.54	−13.32	PBn13^b^	0.16	29.54*
CH I×PBn7	1.42	−8.88	CH I	−0.10	29.14**	PBn7	−2.26	−38.79**

*Significant at P = 0.05;

**Significant at P = 0.01.

a–c indicate same values, included for purpose of explanation. Respective parents were used only once in diallel cross.

#### Components of variance

The genetic component analysis indicated the significance of dominance effects in resynthesized *B. juncea* as well as the progenitor species for both seed as well as the biomass yield (cf. Table 7). Additive gene effects were non-significant for seed yield but significant for biomass yield in resynthesized *B. juncea* and one progenitor species, *B. nigra*. These were non-significant for *B. rapa*. In all the instances, the values for dominance component of variation were higher than those recorded for additive genetic variation. Both the H_1_, component of variance due to dominance effect of gene as well as H_2_, the proportion of dominance variation due to positive (u) and negative (v) effects of the gene, were higher in magnitude than the additive effects. H_2_ was mostly smaller in magnitude than H_1_. Net dominance effects over all the loci in heterozygous phase, indicated by h^2^, were positive and significant for seed yield in resynthesized *B. juncea* and for biomass yield in progenitor *B. nigra*. Excess of dominant alleles was suggested by positive and significant F values in resynthesized *B. juncea* and progenitor species *B. nigra*. For seed yield, F values were positive for *B. juncea* and *B. rapa* but negative in *B. nigra*. E, the variation due to non heritable component associated with individual means was non significant for seed yield in resynthesized *B. juncea* and for both seed yield as well as biomass yield in *B. rapa*. E values were significant for biomass yield in both *B. juncea* and *B. nigra*. Overall, high level of dominance components [H_1_, H_2_] and low magnitude of additive component (D) were indicative of dominant gene effects. These inferences were further supported by low heritability estimates for both seed yield and biomass yield in resynthesized *B. juncea* and progenitor species. Degree of dominance being greater than 1, also suggested over dominance for both the traits in all the three species. Equal proportion of genes with positive and negative effects was observed for seed yield in resynthesized *B. juncea* and *B. nigra*. This proportion was, however, unequal for seed yield in *B. rapa* and for all the three species for biomass yield. Aside the seed yield in *B. nigra*, the proportion of dominant and recessive genes in the parents was higher than 1, suggesting the excess of dominant genes.

**Table 7:** Genetic components and ratio between components in diallel crosses of resynthesized *B. juncea* and progenitor species *B. rapa* and *B. nigra*.

Component	Seed Yield			Biomass Yield		
	*B. juncea*	*B. rapa*	*B. nigra*	*B. juncea*	*B. rapa*	*B. nigra*
D	40.65	110.84	5.84	8848.05*	1490.88	7094.98*
H_1_	1127.44*	584.45*	232.44*	31294.27*	32110.42*	13517.18*
H_2_	1157.37*	426.73*	241.88*	26443.28*	24579.53*	10685.44*
h^2^	402.79*	−27.69	67.64	1995.29	1698.13	7362.75*
F	50.47	217.01	−29.65	9763.25*	4314.73	6098.41*
E	35.50	90.37	43.63*	1523.34*	1517.17	1880.56*
h^2^ (ns)	0.09	0.11	0.10	0.20	0.23	0.30
Degree of dominance	5.50	2.30	6.31	1.88	4.64	1.38
Genes with +/− effects	0.24	0.18	0.25	0.21	0.19	0.20
Genes with dom/rec effects	1.25	2.48	0.43	1.83	1.90	1.90

### Association of hybrid performance and heterosis with genetic diversity, general combining ability and specific combining ability

There was no correlation between hybrid performance and genetic diversity for seed yield in any of the three species evaluated (cf. Table 8). For biomass yield, hybrid performance was correlated with genetic diversity in *B. juncea* and *B. rapa*. No association of genetic distance with absolute mid-parent heterosis ( AMPH) or RMPH occurred in any of the species for seed yield as well as biomass yield. For AMPH as well as RMPH, negative correlation was observed in *B. nigra* for biomass yield. Other combinations showed no correlation. Correlation analysis between GCA and RMPH/RHPH revealed significant association of GCA with heterosis in *B. rapa* for biomass yield only. SCA effects were significant and had positive association with hybrid performance as well as with both kinds of heterosis for seed yield as well as biomass yield. The correlation was uniform across all the three species. Specific combining ability, however, did not depend on the genetic diversity, the correlations being non significant for all the species investigated.

**Table 8:** Correlation in *B. rapa*, *B. nigra* and *B. juncea* for classical heterosis (i) F_1_P and GD, (ii) Heterosis and GD, (iii) Heterosis and GCA, (iv) F_1_P and SCA, (v) Heterosis and SCA and (vi) SCA and GD.

Correlation	Seed Yield			Biomass Yield		
	*B. rapa*	*B. nigra*	*B. juncea*	*B. rapa*	*B. nigra*	*B. juncea*
r(F_1_P, GD)	0.09	−0.09	0.10	0.28*	−0.08	0.35*
r(AMPH,GD)	0.08	−0.07	0.06	0.21	−0.19	0.17
r(RMPH,GD)	0.16	−0.05	0.04	0.21	−0.13	0.16
r(AHPH,GD)	−0.05	−0.10	0.04	0.18	−0.36*	0.07
r(RHPH,GD)	0.04	−0.01	0.06	0.19	−0.27*	0.11
r(AMPH,GCA)	0.05	−0.19	0.17	0.52*	−0.12	−0.11
r(RMPH,GCA)	0.08	−0.16	0.10	0.48*	0.04	−0.13
r(AHPH,GCA)	−0.13	−0.23	0.15	0.44*	−0.30*	−0.27
r(RHPH,GCA)	0.01	−0.20	0.11	0.43*	−0.02	−0.19
r(F_1_P,SCA)	0.90*	0.92*	0.96*	0.74*	0.79*	0.89*
r(AMPH,SCA)	0.88*	0.96*	0.94*	0.89*	0.89*	0.91*
r(RMPH,SCA)	0.83*	0.95*	0.92*	0.88*	0.91*	0.86*
r(AHPH,SCA)	0.85*	0.91*	0.90*	0.89*	0.74*	0.81*
r(RHPH,SCA)	0.84*	0.93*	0.88*	0.89*	0.87*	0.83*
r(SCA, GD)	−0.06	−0.10	0.13	0.07	−0.13	0.20

*Significant at P = 0.05.

### Interplay of alloploidy with genetic diversity, gene action and heterosis

The data from eleven resynthesized *B. juncea* types, their 55 F_1_ combinations, parental *B. rapa* and *B. nigra* genotypes and their respective F_1_ combinations developed through half diallel analysis were used to draw inferences regarding the role of fixed heterosis in the performance of an amphiploid, and to establish correlation between fixed heterosis and classical heterosis. Association of fixed heterosis in amphiploids with the combining ability of the diploid progenitor parents was also studied.

#### Fixed heterosis *vs.* amphiploidy

**Figure 5:**
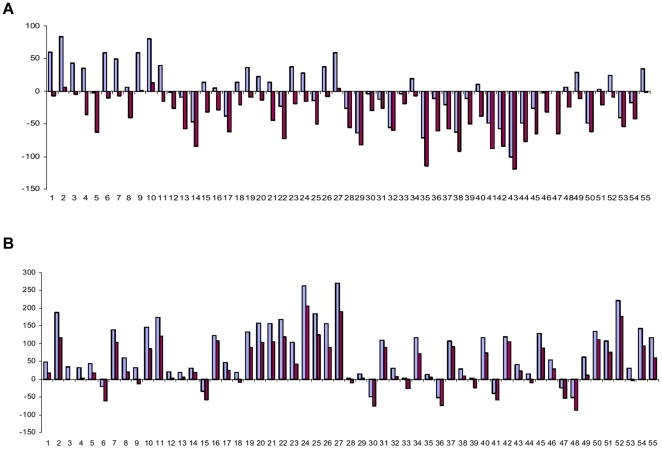
Pictorial representation of the manifestation of fixed heterosis for seed yield (A) and biomass yield (B) in respective cross combinations of *Brassica juncea* over mid- parent (blue) and high parent (red) values.

Data for fixed heterosis for seed yield are presented in Table S1 as well as Figure 5A. Fixed heterosis over the mid parent values ranged from −100.3 (SJR 23×SJR 113( to 83.7 per cent (SJR 2×SJN 8). The corresponding values for the fixed heterosis over high parent (cf.Table S1) were −118.6 (SJR 23×SJR 113) to 13.2 per cent (SJR 2×SJR 63). Average fixed heterosis values over mid parent and high parents were low, being −1.0 and −38.9 per cent respectively. However, very high proportion (0.47) of combinations showed positive values for fixed heterosis over mid parent performance. For biomass yield ( cf. Figure 5B), the fixed heterosis over mid parent value ranged from −51.5 (SJR 17×SJN 14) to very high value of 270.9 (SJN 8×SJR 63). The average fixed heterosis over mid parent was also very high (76.4%). Fixed heterosis over the high parent is presented in Figure 5. It ranged from −87.5 (SJN 14×SJR 57) to 206.5 per cent (SJN 8×SJR 76). The average values for fixed heterosis over the mid parent and high parent were 76.4 and 41.4 per cent respectively.

#### Fixed heterosis *vs.* classical heterosis

**Figure 6:**
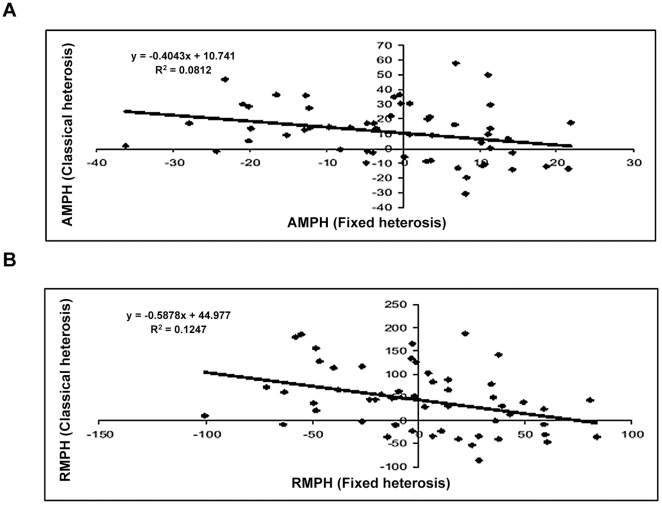
Association between fixed and classical heterosis for resynthesized *Brassica juncea* and progenitor species for seed yield over absolute mid parent (A) and relative mid parent (B) values.

**Figure 7:**
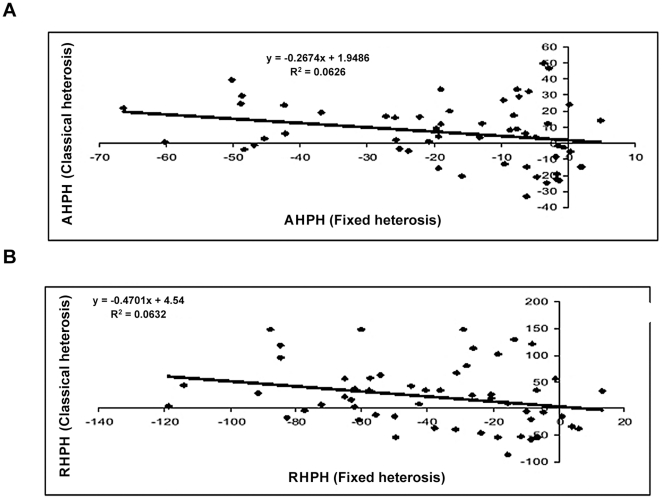
Association between fixed and classical heterosis for resynthesized *Brassica juncea* and progenitor species for seed yield over absolute high parent (A) and relative high parent (B) values.

**Figure 8:**
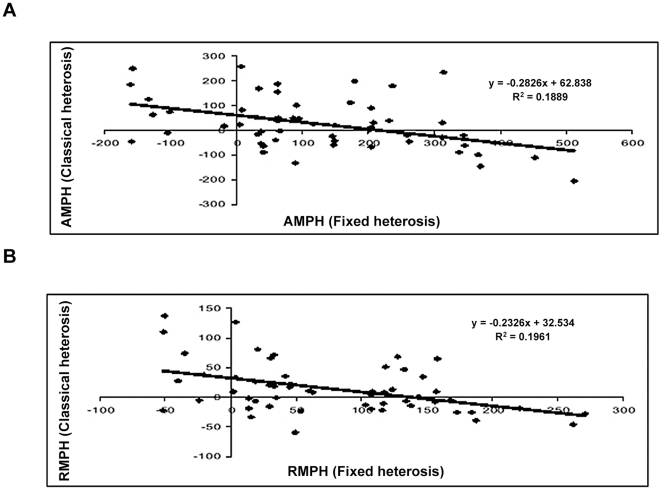
Association between fixed and classical heterosis for resynthesized *Brassica juncea* and progenitor species for biomass yield over absolute mid parent (A) and relative mid parent (B) values.

**Figure 9:**
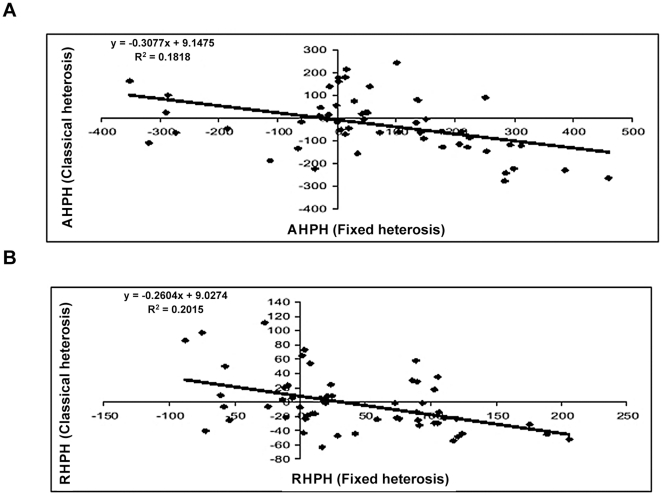
Association between fixed and classical heterosis for resynthesized *Brassica juncea* and progenitor species for biomass yield over absolute high parent (A) and relative high parent (B) values.

The correlation coefficients between fixed heterosis and classical heterosis for both seed yield as well as biomass yield are presented in Table 9. Both the correlations were negative and significant for heterosis over mid parent as well as high parent values. Similar trends were reflected in the regression studies (cf. Figures 6, 7, 8, 9). Attempt was made to compartmentalize overall heterosis in three categories based on genetic diversity (cf. Table 10). The category one comprised crosses involving parents with low genetic diversity (<0.2), the second category included the combinations in the intermediate range (0.2–0.25), while the third category comprised the hybrids having highly diverse parents (>0.25). Except for the intermediate category (0.2–0.25), where no correlation was observed, the trend was largely similar to the overall correlation values.

**Table 9:** Correlation between classical and fixed heterosis for seed and biomass yield in crosses of resynthesized *B. juncea*.

Trait	Heterosis	Genetic Distance			
		<0.2	0.2–0.25	>0.25	Overall
Seed Yield	AMPH	−0.38	−0.16	−0.33*	−0.28*
	RMPH	−0.47	−0.28	−0.38*	−0.34*
	AHPH	−0.51*	−0.05	−0.26	−0.25*
	RHPH	−0.44	−0.12	−0.27	−0.25*
Biomass Yield	AMPH	−0.86*	−0.01	−0.48*	−0.43*
	RMPH	−0.72*	−0.14	−0.50*	−0.44*
	AHPH	−0.95*	−0.17	−0.36*	−0.43*
	RHPH	−0.81*	−0.24	−0.43*	−0.45*

*Significant at P = 0.05.

**Table 10:** Correlation of genetic distance and various kind of heterosis for seed and biomass yield in crosses of resynthesized *B. juncea*.

Trait	Heterosis type	Heterosis	Genetic Distance			
			<0.2(r)	0.2–0.25(r)	>0.25(r)	Overall
Seed Yield	Calssical	AMPH	0.16	−0.33	−0.08	0.06
		RMPH	−0.01	−0.43*	−0.05	−0.04
		AHPH	0.07	−0.42*	−0.06	0.04
		RHPH	0.00	−0.49*	0.00	0.06
	Fixed	AMPH	0.59*	0.59*	0.44*	0.29*
		RMPH	0.55*	0.63*	0.36*	0.27*
		AHPH	0.67*	0.29	0.61*	0.30*
		RHPH	0.66*	0.40*	0.56*	0.32*
Biomass yield	Classical	AMPH	0.58*	−0.41*	−0.22	0.17
		RMPH	0.50*	−0.45*	−0.11	0.16
		AHPH	0.57*	−0.51*	−0.16	0.07
		RHPH	0.51*	−0.53*	−0.16	0.11
	Fixed	AMPH	−0.46	0.18	0.33*	0.03
		RMPH	−0.38	0.31	0.38*	0.11
		AHPH	−0.52*	−0.24	0.32*	0.03
		RHPH	−0.46	0.21	0.38*	0.06

*Significant at P = 0.05.

#### Influence of diploid progenitor species on genetic diversity and heterosis in the allotetraploid species

Genetic diversity in diploid progenitors was not reflected in *B. juncea*. F_1_ performance, general combining ability, specific combing ability and heterosis [both mid parent as well as high parent] observed in *B. rapa* appeared to have no correlation with their corresponding values observed at amphiploid level (cf. Table 8). Classical heterosis for seed as well as biological yield showed no correlation with genetic diversity on average basis (cf. Table 8). Dissection of this correlation at varied levels of genetic diversity (<0.2, 0.2–0.25 and >0.25) indicated negative correlation between classical heterosis and genetic diversity for both seed yield as well as biomass yield in the class with genetic diversity level above .25 (Table 9) . Biomass yield showed negative heterosis in combinations showing low genetic diversity(<0.2). Without any exception, fixed heterosis had positive and significant correlation with genetic diversity on overall basis as well as on the basis of various diversity groupings for seed yield (cf. Table 10). Interestingly, for the biomass yield, the correlation was significant and positive at higher level of genetic diversity (>0.25). No correlation existed at average or intermediate level of diversity([0.2–0.25), while it was mostly negative at low diversity (<0.2). To further elucidate the contribution of genetic diversity to classical heterosis, multiple correlation analysis was conducted using classical heterosis for seed/biomass yield as dependent variable (cf. Table 11). Genetic distance and combining ability of resynthesized *B. juncea* and progenitor species were taken as independent variables along with fixed heterosis for both seed as well as biomass yield. R^2^ values more than or approaching 0.90 were indicative of the optimum choice of the independent variables as contributors for heterosis. The genetic diversity of *B. juncea*, *B. rapa* and *B. nigra* genotypes as well as the specific combining ability in *B. nigra* were the significant contributors to classical heterosis in *B. juncea* combinations for seed as well as biomass yield. Contribution of heterosis and specific combining ability in *B. rapa* were of no significance for over all expression of heterosis. Contribution of fixed heterosis was negative but significant.

**Table 11:** Multiple correlation coefficient (R^2^), constant and partial regression coefficients from multiple regression analysis.

Source	Seed Yield		Biomass Yield	
	AMPH(J)	AHPH(J)	AMPH(J)	AHPH(J)
R^2^	0.95	0.91	0.98	0.84
Constant	6.07	−34.79*	−4.08	34.04
GD (J)	0.58*	0.84*	0.82*	−0.15
SCA (J)	0.41*	0.83E-01	0.20*	1.28*
GD (R)	0.16*	0.23*	0.89E-01*	−0.11
AMPH/AHPH(R)	−0.10	−0.28	−0.11	0.18
SCA (R)	0.40E-01	0.17	0.67E-01	−0.14E-01
GD (N)	−5.81	0.55*	0.60*	0.65*
AMPH/AHPH(N)	−0.63*	0.76*	−0.58*	0.98E-01
SCA (N)	0.56*	−1.40*	−0.48E-01	−0.82*
AMPH/AHPH(Fixed)	−0.35*	−0.35*	−0.49*	−0.34*

## Discussion

Heterosis in interspecific hybrids is permanently fixed in the respective allopolyploids in the event of chromosomes doubling. This is exemplified by many allopolyploids that became self-pollinating irrespective of pollinating patterns in the parents. This type of heterosis is heritable and is selected in the allopolyploid progeny. Studies conducted across genera [23] have indicated that interspecific hybrids generally show more heterosis than intraspecific hybrids, if the genetic difference between the species or genera does not prevent them from forming compatible crosses. The choice of parents of hybrid combinations is normally based on the estimates of genetic diversity to identify heterotic combinations with relatively fewer real time field evaluations. In present communication, genetic diversity based on DNA polymorphism of resynthesized *B. juncea* genotypes and progenitor species *B. rapa*/*B. nigra*, revealed lack of correlation between genetic diversity at amphiploid level and the demonstrated diversity in genotypes of parental diploid progenitor species. This inference is significant as it goes against current concept of using genetically diverse diploid parents in order to produce diverse digenomics. There was also no indication of any relationship between heterosis for seed and biomass yield obsereved at diploid level and that recorded at alloploid level. Theoretical considerations [24] and computer modeling [25], however, suggest that lower correlation between genetic distance and heterosis may also result if the QTLs influencing heterosis are not closely linked to the molecular markers used for diversity analysis.

There is also interest in estimation of genetic components underlying heterosis for quantitative traits. From classical quantitative genetic perspective, such components have been defined as additive and dominance effects to represent the linear and nonlinear effects within a locus and epistasis for deviation from additivity between loci [26]. In a typical one locus and two alleles system, the epistatic effects are further partitioned into additive×additive effect of two loci (AA), additive effect of first locus×dominance effect of the second locus (AD), dominance×dominance effects of two loci (DD) and dominance effect of first locus×additive effect of the second locus (DA). Numerous studies are available for natural *B. juncea* that emphasize the importance of both additive and non additive genetic variance with primacy of former. Role of occasional epistasis for yield and its components have also been demonstrated. We estimated the gene action for seed as well as biomass yield across two ploidy domains. Combining ability analysis revealed highly significant variances for GCA and SCA for both seed and biomass yield in resynthesized *B. juncea* as well as its progenitor species. No correlation existed between general and specific combining ability effects observed at diploid and amphiploid level for seed yield.

Failure of good general/specific combiners at diploid level, to produce better general/specific combiners at amphiploid level may be attributed to dominance whose role in general combining ability has been emphasized in the past [27]. It has been pointed out that in the absence of epistasis, general combining ability comprised both additive and non-additive gene effects and specific combining ability involved dominance effects only. This likely is a case of differentially expressed genes in amphiploid and progenitor species. The changes can occur immediately after allopolyploid formation or stochastically in the selfing progenies [28] and may be mediated by genetic [sequence-dependent], epigenetic [sequence-independent] mechanisms or both. Theoretically, additivity, dominance and epistasis all contribute to the average effect of alleles and the additive genetic variance. The genetic component analysis indicated the significance of dominance variance in *B juncea* and donor diploid species for both seed as well as biomass yield. Its values were higher than those recorded for additive genetic variation. Excess of dominant alleles was observed in *B. nigra* and resynthesized *B. juncea.* These results for resynthesized *B. juncea* are at variance with majority of the studies reported earlier in natural *B. juncea* which underlined the importance of additive gene effects [29]. It seems that additivity is a long term consequence of amphiploidy. In the present context, the diallel crosses were generated utilizing resynthesized *B. juncea* types in early generations following amphiploidy [A_3_]. Aside the seed yield in *B. nigra*, the proportion of dominant and recessive genes in the parents was higher than 1, suggesting the excess of dominant genes besides emphasizing the importance of dominance variation, across the ploidy domains in the test germplasm. Apparently much of the fixed heterosis or heterosis due to amphiploidy could be explained in term of non additivity. High broad sense and low narrow sense estimates of heritability were also suggestive of the fact that high broad sense heritability values were largely due to non-additive variation. Our studies may be seen in the light of gene expression assays. Genome-wide non additive expression of homoeologous loci has been extensively studied in many interspecific hybrids and allopolyploids, including *Arabidopsis*, *Brassica*, cotton, and wheat. The levels of differentially expressed genes between the related species are higher than those within species. Over 15–50% of genes are differentially expressed between the related species. Over 94% of the repressed genes in the allotetraploids are expressed at higher levels in *A. thaliana* than in *A. arenosa*; it is consistent with overall suppression of the *A. thaliana* phenotype in the synthetic allotetraploids and natural *A. suecica* [30]. At gene expression level, hybrid yield and heterosis have been reported [31] to be associated positively with the proportion of additively expressed genes, negatively with the proportion of paternally expressed genes, and not correlated with over or under-expression of some specific genes. No association of genetic distance with heterosis occurred in any of the species for seed yield as well as biomass yield. Correlation studies between GCA and RMPH/RHPH revealed significant association of GCA with heterosis in *B. rapa* for biomass yield only. SCA effects were significant and showed positive association with hybrid performance as well as both kinds of heterosis for seed yield as well as biomass yield. The correlation was uniform across all the three species. Specific combining ability, however, did not depend on the genetic diversity, the correlations being non significant for all the species investigated.

Our studies clearly indicated that fixed heterosis on the basis of population averages was not a significant contributor to the performance of the amphiploid for seed yield; it however, contributed significantly to the biomass yield as almost half of the hybrid combinations showed positive values for fixed heterosis over mid parent performance. There can be several possibilities regarding the adaptive significance of fixed heterosis. One can postulate that fixed heterosis is an important contributor to evolutionary success of amphiploids. High biomass, indeterminacy and more growing points in a de novo amphiploid may endow it with greater competitive ability over cohabiting diploid progenitor species. In comparison, seed yield is a component that largely is a consequence of human selection since it is a manifestation of improved harvest index and determinacy which may or may not have any adaptive importance. It is also possible that heterosis for vegetative growth is different from that for reproductive growth as both might be controlled by different sets of genes and regulatory pathways.

Resynthesis of *B. juncea* and *B. napus* is being carried out across the world to improve germplasm diversity, to increase selection efficiency as well as to expand the limits of classical heterosis. The results as described in the present communication and reported in the past, clearly demonstrate that the increasing the genetic diversity in germplasm may help to improve selection efficiency but is certainly not a definitive method to enhance heterosis in alloploid species. As discussed earlier the genetic diversity was not correlated with classical heterosis in general. Part of the failure of increased genetic diversity to be translated into enhanced classical heterosis can now be ascribed to the fixed heterosis (this communication) as a consequence of alloploidy. This fixed heterosis is negatively associated with classical heterosis for seed as well as biomass yield. The correlation analysis of genetic diversity in diploid progenitors and allotetraploid *B. juncea* indicated absence of association at all the levels of diversity. Apparently genetic diversity in resynthesized alloploid was not dependent upon genetic diversity in any of the progenitor species. We also explored possibility of defining heterosis related general and specific combining abilities at diploid and amphiploid level. F_1_ performance, general combining ability, specific combing ability and heterosis (both mid parent as well as high parent) observed in *B. rapa* appeared to have no correlation with their corresponding values observed at amphiploid level. Very interesting results were, however, obtained for seed yield in case of *B. nigra*. General combining ability as well as heterosis for seed yield in *B. nigra* combinations had a positive and significant association with general combining ability and heterosis in *B. juncea*. This is a very interesting finding as in the past it was believed that *B. rapa* (AA) is the source of productivity traits for *B. juncea* (AABB), while *B. nigra* (BB) was a repository of only the defensive traits. Apparently there are certain unexpressed yield related genetic information in *B. nigra* that is expressed at the amphiploid level. Thus it may be possible to improve the performance of a resynthesized *B. juncea* amphiploid in a hybrid combination based on the choice of *B. nigra* parent(s).

Without any exception, fixed heterosis had positive and significant correlation with genetic diversity on overall basis as well as on the basis of various diversity groupings. For biomass yield there was no correlation with genetic diversity on an average. Interestingly, for the biomass yield, the correlation was significant and positive at higher level of genetic diversity (>0.25). No correlation existed at intermediate level of diversity (0.2–0.25), while it was mostly negative at low diversity (<0.2). Based on above results one can conclude that while genetic diversity *per se* may not be a significant contributor to the classical heterosis, it is an important contributor to the fixed heterosis due largely to the buffering and consequently the adaptive advantage it provides to the *de novo* alloploids during evolution. Multiple correlation analysis was conducted using classical heterosis for seed/biomass yield as dependent variable. Genetic distance and combining ability of resynthesized *B. juncea* and progenitor species were used as independent variables along with fixed heterosis for both seed as well as biomass yield. The genetic diversity of *B. juncea*, *B. rapa* and *B. nigra* genotypes as well as the specific combining ability in *B. nigra* were the significant contributors to classical heterosis in *B. juncea* combinations for seed as well as biomass yield. Contribution of heterosis and specific combining ability in *B. rapa* were of no significance for over all expression of heterosis. Contribution of fixed heterosis was negative but significant. Exclusion of AMPH and SCA from the multiple regression matrixes had no appreciable effect on R^2^ values. Based on this analysis, it may thus be concluded that the genetic diversity may still be a contributor to the expression of classical heterosis if its role is not masked by negative correlation between fixed heterosis and classical heterosis. Mapping populations (RILs) have already been developed in selected combinations for identifying heterosis related QTL's in *B. juncea* and progenitor species and their expression across the ploidy domains.

## Materials and Methods

The present investigations were carried out in the Department of Plant Breeding and Genetics, Punjab Agricultural University, Ludhiana. The basic experimental material comprised core collection of Indian and exotic accessions and landraces of *Brassica rapa* and *B. nigra*, selected on the basis of F_1_ heterosis for productivity traits following field evaluation of a large number of hand bred intervarietal F_1_ hybrids of both species (data not included).The selected genotypes were hybridized to develop eleven resynthesized *B. juncea* genotypes. For further genetic studies, eleven resynthesized *B. juncea* amphiploids (A_3_) were crossed in a half diallel fashion to develop 55 F_1_ combinations. Similarly grandparent genotypes, ten of *B. rapa* and nine of *B. nigra* were also crossed in a half diallel fashion to generate 45 and 36 hybrid combination respectively. These three sets of parental lines along with their respective F_1_ combinations were evaluated separately in randomized block design with two replications. Each replication comprised parental genotypes as well as the F_1_ hybrid combinations raised in paired rows, each row being 3 meter long. Row to row distance of 30 cm and plant to plant distance of 15 cm were maintained. The crop was raised with standard agronomic practices. Data for different agronomic traits was recorded on ten random plants per genotype/F_1_ in each replication but both the rows were bulk harvested to measure seed and above ground biomass yield.

### Genetic diversity

Simple Sequence Repeats (SSR) primers were used for assaying genotypic diversity within *B. rapa*, *B. nigra* germplasm lines and resynthesized *B. juncea*. Protocol as described by Shokeen [32] was utilized to generate SSR based DNA polymorphism. DNA was isolated by using standard procedure [33]. Genetic diversity was estimated on the basis of polymorphism generated by SSR primers. Amplification profiles of test genotypes were compared with each other and bands of DNA fragments were scored as ‘1’ for present , ‘0’ for absent and ‘9’ in case of no amplification. A common set of forty six A-genome or B-genome specific primers [34], [35] were used to assay genetic diversity. The data for all primers were compiled and used to estimate the genetic distance in the form of Modified Rogers Distance([MRD ) between pairs of parental lines [36]. The genetic distance i.e. MRD was computed using computer programme NTSYS_PC_Ver 2.02e [37]. The data from all primes were used to estimate the similarity on the basis of the number of shared amplified bands. Similarity was calculated with SIMQUAL function of NTSYS programme, which computed a variety of similarity and dissimilarity coefficients (association coefficients) for qualitative data. The similarity matrix value[s] based on DICE coefficient of similarity was calculated and used to develop a dendrogram. Genetic distance (MRD) was calculated from the similarity coefficient by subtracting it from one.

### Statistical analysis

Mean values of the parents and F_1_'s in each replication for different characters were used for statistical analysis. The data obtained for seed and biomass yield were subjected to the analysis of variance by the method of randomized complete block design which is based on the linear model [38]. Combining ability, component of variance, and heterosis were also estimated.

#### Heterosis

Mid parent [RMPH/AMPH] and high parent (RHPH/AHPH) heterosis were estimated after Matzinger [39]. Fixed heterosis or the heterosis due to amphiploidy was first calculated for each resynthesized *B. juncea* by the formula:
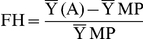
Where,
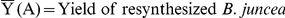


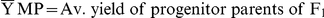


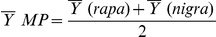
High parent heterosis was also similarly obtained, by replacing the value of MP with value of better performing parent (HP) in the equation.

#### Combining ability analysis

General and specific combining ability effects and variances were computed by Griffing's method 2 (parents plus all possible one way crosses i.e. a = (p (p+1)/2) using Fixed effect model [40]. Components of variance were computed by the use of equations given by Jinks [41] and Hayman [42].

#### Correlation coefficient (r), Regression and multiple correlations

These were calculated using standard statistical software's. Heterosis over mid parent and high parent values in all cross combinations of resynthesized *B. juncea* were correlated with MRD, SCA, GCA, *per se* performance of parental *B. juncea* and their progenitor species genotypes to assess the extent of correlation between these genetic parameters and parental diversity.

## Supporting Information

Table S1 **Fixed and classical heterosis in respective cross combinations of resynthesized** ***B. juncea*** **for seed yield and biomass yield over mid parent and high parent values.**
(DOCX)
