# Supplementary material for: Heterosis as Investigated in Terms of Polyploidy and Genetic Diversity Using Designed Brassica juncea Amphiploid and Its Progenitor Diploid Species
Source: PLoS One. 2012 Feb 21;7(2):e29607. doi: 10.1371/journal.pone.0029607 (PMC3283606; doi:10.1371/journal.pone.0029607)
Supplement: Table S1 — Fixed and classical heterosis in respective cross combinations of resynthesized B. juncea for seed yield and biomass yield over mid parent and high parent values. (DOCX) [file pone.0029607.s001.docx]

**Table S1 : Fixed and classical heterosis in respective cross combinations of resynthesized *B. juncea* for seed yield and biomass yield over mid parent and high parent values**

|  |  | **Yield Heterosis** | | | | **Biomass Heterosis** | | | |
| --- | --- | --- | --- | --- | --- | --- | --- | --- | --- |
| **Code** | **Resynthesized *B. juncea*** | **RMPH(F)** | **RMPH (C)** | **RHPH(F)** | **RHPH (C)** | **RMPH(F)** | **RMPH (C)** | **RHPH(F)** | **RHPH (C)** |
|  |  |  |  |  |  |  |  |  |  |
| SJR2 x SJR59 | (Sunford x Pak) x (EC513427xPBn22) | 60.3 | -46.2 | -6.5 | -53.8 | 49.1 | -58.9 | 17.3 | -63.0 |
| x SJN8 | (PBn17 x EC523426) | 83.7 | -35.8 | 6.1 | -37.8 | 187.6 | -38.5 | 117.1 | -54.1 |
| x SJR6 | (Sunbean x PBn13) | 43.1 | 14.2 | -4.5 | -6.8 | 34.6 | -0.9 | 0.0 | -7.0 |
| x SJR17 | (TCN01 x PBn16) | 35.3 | 50.8 | -36.0 | 33.9 | 33.1 | 72.1 | 2.3 | 65.7 |
| x SJR23 | (Sonja x PBn16) | -1.9 | 53.9 | -63.1 | 16.6 | 44.8 | 17.4 | 16.2 | 6.1 |
| x SJN14 | (PBn12. x Torch) | 58.7 | 24.5 | -9.6 | -4.3 | -20.7 | 38.5 | -60.9 | 9.4 |
| x SJR76 | (EC513427 x PBn19) | 49.2 | 39.5 | -6.6 | 34.3 | 138.0 | -13.8 | 103.1 | -29.4 |
| x SJR113 | (EC 101 x PBn20) | 6.6 | 83.8 | -40.5 | 34.3 | 60.4 | 10.5 | 21.1 | 9.0 |
| x SJR57 | (Mitra x PBn13) | 58.6 | -8.4 | 1.0 | -13.9 | 33.3 | 18.3 | -12.9 | 3.8 |
| x SJR63 | (CH I x PBn7) | 80.5 | 44.2 | 13.2 | 32.7 | 147.1 | 34.9 | 86.2 | 30.1 |
| SJR59 x SJN8 | (EC27 x PBn22)x (PBn17 x EC523426) | 39.2 | 31.1 | -15.4 | 9.6 | 173.4 | -24.9 | 120.8 | -47.7 |
| x SJR6 | (Sunbean x PBn13) | -1.4 | 126.5 | -25.9 | 112.9 | 20.4 | 81.0 | 3.7 | 73.0 |
| x SJR17 | (TCN01 x PBn16) | -9.2 | 63.6 | -57.4 | 57.5 | 18.9 | -6.4 | 5.9 | -18.5 |
| x SJR23 | (Sonja x PBn16) | -46.4 | 128.3 | -84.6 | 96.1 | 30.6 | 20.1 | 19.9 | -1.1 |
| x SJN14 | (PBn12. x Torch) | 14.2 | 88.7 | -31.0 | 66.8 | -34.9 | 73.9 | -57.3 | 50.0 |
| x SJR76 | (EC513427 x PBn19) | 4.7 | 103.5 | -28.1 | 80.7 | 123.8 | 13.3 | 106.8 | -14.5 |
| x SJR113 | (EC 101 x PBn20) | -37.9 | 66.2 | -61.9 | 36.5 | 46.3 | 22.5 | 24.7 | 9.0 |
| x SJR57 | (Mitra x PBn13) | 14.1 | 31.5 | -20.4 | 19.3 | 19.1 | 26.7 | -9.2 | 23.3 |
| x SJR63 | (CH I x PBn7) | 36.0 | 0.5 | -8.3 | -19.4 | 133.0 | 47.6 | 89.8 | 28.7 |
| SJN8 x SJR6 | (PBn17xEC523426)x (Sunbean x PBn13) | 22.0 | 188.9 | -13.4 | 130.0 | 158.3 | 64.8 | 103.5 | 17.9 |
| x SJR17 | (TCN01 x PBn16) | 14.1 | 65.6 | -44.9 | 43.0 | 156.8 | -7.2 | 105.7 | -29.0 |
| x SJR23 | (Sonja x PBn16) | -23.0 | 45.4 | -72.0 | 7.8 | 168.5 | -4.7 | 119.7 | -23.4 |
| x SJN14 | (PBn12 x Torch) | 37.6 | -39.4 | -18.5 | -54.0 | 103.0 | -12.8 | 42.5 | -44.0 |
| x SJR76 | (EC513427 x PBn19) | 28.1 | -85.4 | -15.6 | -86.4 | 261.7 | -45.6 | 206.5 | -51.9 |
| x SJR113 | (EC 101 x PBn20) | -14.6 | -35.3 | -49.4 | -53.6 | 184.2 | -25.6 | 124.5 | -44.0 |
| x SJR57 | (Mitra x PBn13) | 37.5 | 143.0 | -7.9 | 121.8 | 157.0 | 9.2 | 90.6 | -25.1 |
| x SJR63 | (CH I x PBn7) | 59.4 | -30.1 | 4.3 | -33.8 | 270.9 | -28.0 | 189.6 | -45.0 |
| SJR6 x SJR17 | (Sunbean x PBn13) x (TCN01 x PBn16) | -26.5 | -2.2 | -55.5 | -11.3 | 3.8 | 33.4 | -11.4 | 20.9 |
| x SJR23 | (Sonja x PBn16) | -63.6 | -8.2 | -82.6 | -16.7 | 15.5 | -33.8 | 2.5 | -43.5 |
| x SJN14 | (PBn12. x Torch) | -3.1 | 165.3 | -29.0 | 148.5 | -50.0 | 137.7 | -74.6 | 97.4 |

|  |  | **Yield Heterosis** | | | | **Biomass Heterosis** | | | |
| --- | --- | --- | --- | --- | --- | --- | --- | --- | --- |
| **Code** | **Resynthesized *B. juncea*** | **RMPH(F)** | **RMPH (C)** | **RHPH(F)** | **RHPH (C)** | **RMPH(F)** | **RMPH (C)** | **RHPH(F)** | **RHPH (C)** |
| x SJR76 | (EC513427 x PBn19) | -12.5 | 48.2 | -26.1 | 24.7 | 108.7 | 9.9 | 89.4 | -14.4 |
| x SJR113 | (EC 101 x PBn20) | -55.2 | 186.7 | -59.9 | 148.0 | 31.2 | 66.1 | 7.4 | 53.9 |
| x SJR57 | (Mitra x PBn13) | -3.2 | 134.8 | -18.4 | 101.6 | 4.0 | 127.0 | -26.5 | 111.3 |
| x SJR63 | (CH I x PBn7) | 18.8 | -39.5 | -6.3 | -53.7 | 117.9 | 8.7 | 72.5 | -1.3 |
| SJR17x SJR23 | (TCN01 x PBn16) x (Sonja x PBn16) | -71.4 | 73.4 | -114.1 | 44.3 | 14.0 | -18.0 | 4.8 | -23.3 |
| x SJN14 | (PBn12 x Torch) | -10.9 | -7.9 | -60.6 | -21.3 | -51.5 | -21.7 | -72.4 | -39.8 |
| x SJR76 | (EC513427 x PBn19) | -20.4 | 45.9 | -57.6 | 34.1 | 107.2 | -20.0 | 91.7 | -32.5 |
| x SJR113 | (EC 101 x PBn20) | -63.0 | 61.3 | -91.5 | 28.6 | 29.6 | -14.6 | 9.6 | -16.7 |
| x SJR57 | (Mitra x PBn13) | -11.0 | -9.2 | -50.0 | -14.6 | 2.5 | 10.0 | -24.3 | -6.5 |
| x SJR63 | (CH I x PBn7) | 10.9 | -22.2 | -37.8 | -35.7 | 116.3 | -22.1 | 74.7 | -22.2 |
| SJR23x SJN14 | (Sonja x PBn16) x (PBn12 x Torch) | -48.2 | 156.4 | -87.7 | 148.0 | -39.8 | 27.8 | -58.4 | -6.1 |
| x SJR76 | (EC513427x PBn19) | -57.7 | 181.0 | -84.8 | 118.7 | 118.9 | 51.3 | 105.6 | 35.3 |
| x SJR113 | (EC 101 x PBn20) | -100.3 | 10.6 | -118.6 | 4.8 | 41.4 | 35.9 | 23.6 | 24.4 |
| x SJR57 | (Sonja x PBn16 )x (Mitra x PBn13) | -48.3 | 21.2 | -77.1 | -4.0 | 14.2 | -1.7 | -10.4 | -20.8 |
| x SJR63 | (CH I x PBn7) | -26.4 | 117.4 | -65.0 | 55.8 | 128.1 | 69.0 | 88.7 | 57.9 |
| SJN14x SJR76 | (PBn12 x Torch) x (EC513427x PBn19) | -2.9 | -22.3 | -31.2 | -38.5 | 53.4 | -23.4 | 28.5 | -47.5 |
| x SJR113 | (EC 101 x PBn20) |  |  | -65.0 | 21.6 | -24.2 | -4.5 | -53.6 | -25.3 |
| x SJR57 | (Mitra x PBn13) | 6.5 | -32.9 | -23.5 | -45.9 | -51.3 | 110.8 | -87.5 | 86.2 |
| x SJR63 | (CH I x PBn7) | 28.4 | -33.8 | -11.4 | -51.9 | 62.6 | 8.7 | 11.6 | -16.4 |
| SJR76x SJR113 | (EC27 x PBn19) x (EC 101 x PBn20) | -49.0 | 37.9 | -62.1 | 3.3 | 134.5 | -6.1 | 110.5 | -22.3 |
| x SJR57 | (Mitra x PBn13) | 3.0 | 29.4 | -20.6 | 26.3 | 107.4 | 4.1 | 76.5 | -22.9 |
| x SJR63 | (CH I x PBn7) | 24.9 | -51.9 | -8.5 | -57.3 | 221.2 | -18.4 | 175.6 | -31.2 |
| SJR113x SJR57 | (EC 101 x PBn20) x (Mitra x PBn13) | -39.7 | 113.6 | -54.4 | 62.7 | 29.9 | 21.5 | -5.5 | 5.5 |
| x SJR63 | (CH I x PBn7) | -17.8 | 57.4 | -42.3 | 9.2 | 143.7 | 0.8 | 93.6 | -1.5 |
| SJR57 x SJR63 | (Mitra x PBn13) x (CH I x PBn7) | 34.3 | 79.3 | -0.8 | 56.0 | 116.6 | -10.5 | 59.6 | -23.8 |
|  | **Mean** | **1.0±5.53** | **44.0±9.2** | **-38.9±4.3** | **22.6±8.0** | **76.4±** **10.7** | **14.8±** 5.6 | **41.4±9.3** | **-1.8** **±5.4** |
|  | **Range** | **-100.3**  **– 83.7** | **-85.4 – 188.9** | **-118.6 – 13.2** | **-86.4 – 153.7** | **-51.5 – 270.9** | **-58.89 – 137.68** | **-87.5 – 206.5** | **-63.0**  **– 111.28** |

***F and C in parenthesis stands for fixed and classical heterosis respectively**
